# Supplementary material for: Resolving the full spectrum of human genome variation using Linked-Reads
Source: Genome Res. 2019 Apr;29(4):635–45. doi: 10.1101/gr.234443.118 (PMC6442396; doi:10.1101/gr.234443.118)
Supplement: Supplemental Material [file supp_gr.234443.118_Supplemental_longranger-2.2.zip › longranger-2.2.tar.bz2!/lr2.2-public/lib/go/src/code.google.com/vitess/docs/user-guide/schema-swap.html]

Vitess / Schema Swap


Toggle navigation

- Overview
- Guides
- Reference
- Blog
- GitHub

- #### Overview

  - What is Vitess
  - Scaling MySQL with Vitess
  - Key Concepts
- #### Getting Started

  - Run Vitess on Kubernetes
    - Custom Docker Build
  - Run Vitess Locally
- #### User Guide

  - Introduction- Client Libraries- Backing Up Data- Reparenting
        - Schema Management
        - VSchema Guide
        - Schema Swap (Tutorial)
        - Sharding
          - Horizontal Sharding (Tutorial, automated)
          - Horizontal Sharding (Tutorial, manual)
          - Sharding in Kubernetes (Tutorial, automated)
          - Sharding in Kubernetes (Tutorial, manual)
        - Vitess Sequences
        - MySQL Server Protocol
        - Vitess and Replication
        - Update Stream
        - Row Based Replication
        - Topology Service
        - Transport Security Model
        - Launching
          - Scalability Philosophy
          - Production Planning
          - Server Configuration
          - 2PC Guide
          - Troubleshooting
        - Upgrading
- #### Reference Guides

  - Vitess API- vtctl Commands
- #### Other Resources

  - Presentations- Blog- Roadmap
- #### Contributing

  - Contributing to Vitess- GitHub Workflow- Code Reviews
- GitHub

# Schema Swap

- #### Overview

  - What is Vitess
  - Scaling MySQL with Vitess
  - Key Concepts
- #### Getting Started

  - Run Vitess on Kubernetes
    - Custom Docker Build
  - Run Vitess Locally
- #### User Guide

  - Introduction- Client Libraries- Backing Up Data- Reparenting
        - Schema Management
        - VSchema Guide
        - Schema Swap (Tutorial)
        - Sharding
          - Horizontal Sharding (Tutorial, automated)
          - Horizontal Sharding (Tutorial, manual)
          - Sharding in Kubernetes (Tutorial, automated)
          - Sharding in Kubernetes (Tutorial, manual)
        - Vitess Sequences
        - MySQL Server Protocol
        - Vitess and Replication
        - Update Stream
        - Row Based Replication
        - Topology Service
        - Transport Security Model
        - Launching
          - Scalability Philosophy
          - Production Planning
          - Server Configuration
          - 2PC Guide
          - Troubleshooting
        - Upgrading
- #### Reference Guides

  - Vitess API- vtctl Commands
- #### Other Resources

  - Presentations- Blog- Roadmap
- #### Contributing

  - Contributing to Vitess- GitHub Workflow- Code Reviews

# Schema Swap: A Tutorial

This page describes how to apply long-running schema changes in Vitess/MySQL
without disrupting ongoing operations. Examples for long-running changes on
large databases are `ALTER TABLE` (for example to add a column), `OPTIMIZE
TABLE` or large-scale data changes (e.g. populating a column or clearing out
values).

If a schema change is not long-running, please use the simpler vtctl
ApplySchema instead.

## Overview

One solution to realize such long-running schema changes is to use a temporary
table and keep it in sync with triggers as originally proposed by
Shlomi
and further refined by others (Percona's
pt-online-schema-change,
Square's Shift).

Here we describe an alternative solution which uses a combination of MySQL's
statement based replication and backups to apply the changes to all tablets.
Since the long-running schema changes are applied to an offline tablet, ongoing
operations are not affected. We called this process **schema swap** due to the
way it's done, and therefore we refer to it by this name throughout the
document.

This tutorial outlines the necessary steps for a schema swap and is based on the
Vitess Kubernetes Getting Started Guide.

**At the high level, a schema swap comprises the following phases:**

1. Apply the schema changes to an offline tablet.
2. Let the tablet catch up and then create a backup of it.
3. Restore all remaining tablets (excluding the master) from the backup.
4. Failover the master to a replica tablet which has the new schema. Restore
   the old master from the backup.
5. At this point, all tablets have the new schema and you can start using it.

**You may be wondering: Why does this work?**

The key here is that the new schema is backward compatible with respect to
statements sent by the app. The replication stream remains backward compatible
as well because we use statement based replication. As a consequence, the new
schema must not be used until it has been changed on all tablets. If the schema
would have been used e.g. when an insert uses a new column, replication would
break on tablets which have the old schema. Swapping schema on all tablets first
ensures this doesn't happen.

Also note that the changes are applied to only one tablet and then all other
tablets are restored from the backup. This is more efficient than applying the
long-running changes on every single tablet.

Now let's carry out an actual schema swap based on our Guestbook example schema.
We'll add a column to it.

## Prerequisites

We assume that you have followed the Vitess Kubernetes Getting Started
Guide up to and including the step "9.
Create a table".

## Schema Swap Steps

1. Got to the Workflows section of vtctld UI (it will be at
   http://localhost:8001/api/v1/proxy/namespaces/default/services/vtctld:web/app2/workflows
   if you followed the Getting Started Guide as is) and press the "+" button in
   the top right corner. You will be presented with "Create a new Workflow"
   dialog.
2. In the "Factory Name" list select "Schema Swap".
3. In the field "Keyspace" enter "test\_keyspace" (without quotes).
4. In the field "SQL" enter the statement representing the schema change you
   want to execute. As an example we want to execute statement "ALTER TABLE
   messages ADD views BIGINT(20) UNSIGNED NULL".
5. Click "Create" button at the bottom of the dialog.

Another way to start the schema swap is to execute vtctlclient command:

```
vitess/examples/local$ ./lvtctl.sh WorkflowCreate schema_swap -keyspace=test_keyspace -sql='SQL statement'
```

From this point on all you need to do is watch how the schema swap process is
progressing. Try expanding the displayed nodes in vtctld UI and look at the logs
of all the actions that process is doing. Once the UI shows "Schema swap is
finished" you can start using the new schema, it will be propagated to all
tablets.

Contact: vitess@googlegroups.com  **·**  
Announcements  **·**  
© 2017 Vitess powered by Google Inc
